# Sensor-Based Glucose Metrics during Different Diet Compositions in Type 1 Diabetes—A Randomized One-Week Crossover Trial

**DOI:** 10.3390/nu16020199

**Published:** 2024-01-08

**Authors:** Kasper B. Kristensen, Ajenthen G. Ranjan, Olivia M. McCarthy, Richard M. Bracken, Kirsten Nørgaard, Signe Schmidt

**Affiliations:** 1 Copenhagen University Hospital—Steno Diabetes Center Copenhagen, 2730 Herlev, Denmark; ajenthen.ranjan@regionh.dk (A.G.R.); olivia.mccarthy@regionh.dk (O.M.M.); kirsten.noergaard@regionh.dk (K.N.); signe.schmidt@regionh.dk (S.S.); 2 Department of Clinical Medicine, Faculty of Health and Medical Sciences, University of Copenhagen, 2200 Copenhagen N, Denmark; 3 Applied Sport, Technology, Exercise and Medicine Research Centre, Swansea University, Swansea SA1 8EN, UK; r.m.bracken@swansea.ac.uk

**Keywords:** glucose management, high fat, high protein, insulin therapy, low carbohydrate, macronutrient composition, type 1 diabetes

## Abstract

By reducing carbohydrate intake, people with type 1 diabetes may reduce fluctuations in blood glucose, but the evidence in this area is sparse. The aim of this study was to investigate glucose metrics during a one-week low-carbohydrate-high-fat (HF) and a low-carbohydrate-high-protein (HP) diet compared with an isocaloric high-carbohydrate (HC) diet. In a randomized, three-period cross-over study, twelve adults with insulin-pump-treated type 1 diabetes followed an HC (energy provided by carbohydrate: 48%, fat: 33%, protein: 19%), HF (19%, 62%, 19%), and an HP (19%, 57%, 24%) diet for one week. Glucose values were obtained during intervention periods using a Dexcom G6 continuous glucose monitoring system. Participant characteristics were: 33% females, median (range) age 50 (22–70) years, diabetes duration 25 (11–52) years, HbA1c 7.3 (5.5–8.3)% (57 (37–67) mmol/mol), and BMI 27.3 (21.3–35.9) kg/m^2^. Glycemic variability was lower with HF (30.5 ± 6.2%) and HP (30.0 ± 5.5%) compared with HC (34.5 ± 4.1%) (P_HF-HC_ = 0.009, P_HP-HC_ = 0.003). There was no difference between groups in mean glucose (HF: 8.7 ± 1.1, HP: 8.2 ± 1.0, HC: 8.7 ± 1.0 mmol/L, P_Overall_ = 0.08). Time > 10.0 mmol/L was lower with HP (22.3 ± 11.8%) compared with HF (29.4 ± 12.1%) and HC (29.5 ± 13.4%) (P_HF-HP_ = 0.037, P_HC-HP_ = 0.037). In conclusion, a one-week HF and, specifically, an HP diet improved glucose metrics compared with an isocaloric HC diet.

## 1. Introduction

It is estimated that 8.4 million people worldwide are living with type 1 diabetes [1]. Despite improvements in the treatment of type 1 diabetes—advanced insulin pumps, glucose monitoring systems, better and faster-acting insulins [2]—only one fifth of adults with type 1 diabetes reach the recommended glycemic target of an HbA1c <53 mmol/mol (<7.0%) [3]. Reaching a glycemic target is crucial to prevent and delay the development of diabetic late complications [4,5]. In addition, the recommendation for people using continuous glucose monitoring is to spend >70% of time with sensor glucose values in the range (TIR) (3.9–10.0 mmol/L), <25% of time above the range (TAR) (>10.0 mmol/L), and <4% time below the range (TBR) (glucose <3.9 mmol/L) [6,7]. Further, the glycemic variability expressed as the coefficient of variation should not exceed 36% [6].

Exogenous insulin and food are the two major determinants of blood glucose in type 1 diabetes. The three most important sources of energy are carbohydrates, fat, and protein, of which the former have the greatest impact on blood glucose. However, there is no evidence suggesting an ideal ratio between these three macronutrients for people living with diabetes. The general recommendation from the American Diabetes Association is that the macronutrient distribution should be based on an individualized assessment of current eating patterns, preferences, and metabolic goals [8].

Despite the lack of specific guidelines regarding dietary macronutrient composition, many people with type 1 diabetes choose to restrict dietary carbohydrate content [9]. There is no universal definition of a low-carbohydrate diet, but the following definitions have been suggested based on a literature review: a low-carbohydrate diet contains less than 130 g of carbohydrate per day, corresponding to less than 26% of total daily energy coming from carbohydrates and a high-carbohydrate diet contains more than 280 g of carbohydrate per day corresponding to more than 55% of total daily energy coming from carbohydrates [9]. For comparison the average carbohydrate intake in an adult population of people with insulin-pump-treated type 1 diabetes in the United Kingdom has been shown to be 166 g per day [10]. However, despite the popularity of low-carbohydrate diets, evidence documenting their effects is limited [9,11]. Short-term studies (1 to 12 weeks) in people with HbA1c values close to target (6.8–7.5% (51–58 mmol/mol)) have demonstrated increased TIR, decreased TBR, and reduction in the coefficient of variation but similar mean glucose with carbohydrate-restricted diets compared with unrestricted diets [12,13,14,15]. The additional potential benefits of a low-carbohydrate diet may include reduced insulin requirements and weight loss [13,14]. On the other hand, concerns regarding low-carbohydrate diet diets include risk of dyslipidemia, hypoglycemia, and diabetic ketoacidosis [9,16].

The beneficial glycemic effects associated with low-carbohydrate diets can be attributed to various factors. For instance, carbohydrate is the macronutrient that induces the fastest and highest postprandial spikes in blood glucose levels [17]. Thus, by reducing carbohydrate consumption, glucose excursions may be mitigated. Furthermore, individuals with type 1 diabetes frequently encounter difficulties in accurately determining the carbohydrate content of their meals, particularly when the content is substantial [18,19]. A 10% estimation error is greater in absolute terms when meal carbohydrate intake is higher as opposed to lower. Accordingly, by lowering carbohydrate intake, the risk of mismatch between food and insulin is minimized.

If carbohydrate intake is reduced, dietary fat and/or protein content need to be increased to meet calorie needs. However, little is known about the effects of substituting carbohydrates with fat and protein, respectively [12]. 

Thus, the aim of this study was to compare blood glucose values during three isocaloric diet types: two different low-carbohydrate diets—low-carbohydrate-high-fat (HF) and low-carbohydrate-high-protein (HP)—and a high-carbohydrate (HC) diet. Secondarily, we explored the impact of the three diets on cardiovascular risk markers.

## 2. Materials and Methods

### 2.1. Ethics and Study Design 

This was a randomized, one-week, three-period, crossover study involving 12 adults with type 1 diabetes. The study was approved by the Regional Committee on Health Research Ethics (H-21042230) and the Danish Data Protection Agency (P-2021-826) and was carried out in accordance with the principles of the Declaration of Helsinki and registered at clinicaltrials.gov (registration no. NCT05268705).

### 2.2. Screening and Randomization Procedures

Participants were recruited from the Steno Diabetes Center Copenhagen, Denmark, and screened for eligibility after having provided informed consent. The main inclusion criteria were: age ≥ 18 years, duration of type 1 diabetes ≥ 5 years, insulin pump use ≥1 year, use of intermittently scanned or continuous glucose monitoring (isCGM/CGM) ≥3 months, HbA1c ≤8.5%, self-reported hypoglycemia awareness, and exercising at least 30 min at moderate or vigorous intensity twice per week. The main exclusion criteria were: use of drugs affecting glucose metabolism (other than insulin) during the study or within 30 days prior to study start, use of a hybrid closed-loop system, ischemic heart disease, severe asthma, pregnancy, or breastfeeding.

At screening, data on diabetes characteristics, anthropometrics, blood pressure, and heart rate were collected. Eligible participants were subsequently randomized using the electronic data capture system, REDCap (Version 13.1.27, Vanderbilt University, Nashville, TN, USA).

### 2.3. Pre- and Post-Intervention Visits 

Within one week prior to and on the first day after each intervention, the participants met in the morning in a fasted state for blood (creatinine, total cholesterol, high-density lipoprotein (HCL), low-density lipoprotein (LDL), very low-density lipoprotein (VLDL), and triglycerides) and urine (u-albumin–creatinine ratio) sampling as well as rested measurements of blood pressure, heart rate, and body weight.

### 2.4. Composition of the Diets

Three diet plans were developed by a registered dietitian for each participant based on the official Danish Dietary Guidelines [13], individual preferences, and estimated energy needs (Supplementary Materials Tables S1–S3 show examples of the three diet plans). The macronutrient content of the diets was defined according to carbohydrate (HF and HP: maximum 100 g/day, HC: minimum 250 g/day) and protein (HC and HF: 1.4 g/kg body weight/day, HP: 1.8 g/kg body weight/day). To achieve isocaloric content of the diets, the amount of fat was determined by the carbohydrate and protein content (Table 1 shows an example of the macronutrient composition of the diets for a 70 kg person). The HC and HF diets were characterized by a relatively high protein content, while the HP diet had a relatively high fat content, albeit lower than that of the HP and HF diets, respectively (see Table 1). Each plan allowed participants to substitute dishes with alternatives, providing dietary variation while still adhering to the diet criteria. As an example, 120 g of potatoes for dinner could be replaced by 75 g of pasta or 45 g of whole-grain bread (Supplementary Materials Table S2).

Participants were instructed to handle cases of hypoglycemia (sensor glucose <3.9 mmol/L) according to their usual procedures. Carbohydrates consumed for hypoglycemia treatment should be entered into the insulin pump, included in the total daily carbohydrate count, and if needed, meal carbohydrate content should be reduced to meet the carbohydrate limits. 

### 2.5. Interventions

During the diet interventions, participants wore a CGM (Dexcom G6, Dexcom Inc., San Diego, CA, USA) and an activity sensor (ActiGraph, wGT3X-BT, Pensacola, FL, USA). The CGM was set to alarm at 3.9 mmol/L. Consistency in activity levels was encouraged among participants throughout all three intervention periods. Further, participants were instructed to utilize the insulin pump bolus calculator for all carbohydrate intakes, regardless of whether insulin administration was required. If necessary, the insulin pump settings were optimized prior to the first diet intervention based on 14-day isCGM or CGM data. We did not specifically advice participants to administer insulin based on protein or fat content, but the participants were allowed to use different bolus types (immediate delivery, delivery over an extended period, and combined boluses with X% of the total bolus as immediate delivery and Y% over an extended period) if they found it appropriate. Likewise, we did not provide specific guidance on the interval between bolus administration and meal start.

Throughout the diet intervention, the primary investigator maintained regular contact with the participants by telephone and encouraged them to reach out if they encountered difficulties following the diet or if they were experiencing any other diabetes-related issues. Between the intervention periods there was a washout period of 5–35 days in which there were no diet restrictions. 

### 2.6. User Involvement

Prior to the study start the dietitian prepared a proposal for the composition of the three different diets. Subsequently, three persons with type 1 diabetes (two females and one male) were invited to provide feedback on the proposals, assessing the feasibility and practicality of following the suggested diets over a 7-day period. Afterwards the dietitian was provided with the participants’ feedback to optimize the design of the diets.

### 2.7. Statistical Analysis

To compare continuous data, such as glucose metrics, a linear mixed-effects model was used. In cases where the data did not follow a normal distribution even after applying logarithmic transformation, the Friedman’s test was applied. For binary outcomes, a generalized linear mixed model was utilized. To account for multiple comparisons, *p*-values for pairwise group comparisons were adjusted using the Tukey–Kramer method. All statistical analyses were conducted using SAS 9.4 (SAS Institute, Cary, NC, USA).

## 3. Results

### 3.1. Participants

From February 2022 to November 2022, 12 participants (four females, of whom three were post-menopausal) were enrolled in and completed the study (Supplementary Materials Figure S1). There were no drop-outs. Baseline characteristics were (median (range)) age of 50 (22–70) years with a diabetes duration of 25 (11–52) years. The median HbA1c level was HbA1c 7.3 (5.5–8.3) % (57 (37–67) mmol/mol) and BMI was 27.3 (21.3–35.9) kg/m^2^. Their usual carbohydrate intake was 166 (62–263) g/day, the total daily basal insulin dose was 19.3 (8.1–72.6) U, and total daily bolus insulin use was 17.8 (8.0–54.4) U. Finally, systolic blood pressure was 142 (131–155) mmHg, diastolic blood pressure was 86 (71–94) mmHg, and pulse was 68 (52–95) bpm. Nine people were using insulin aspart (Novo Nordisk A/S, Bagsværd, Denmark) in their insulin pumps, while three people used faster insulin aspart (Novo Nordisk A/S, Bagsværd, Denmark). Six participants used an Omnipod, four used a Metronic Minimed 640G, one used a Tandem t:slim X2 with basal IQ, and one used an YpsoPump. Nine participants had retinopathy, two participants had nephropathy, and one participant had neuropathy. Six participants were using antihypertensives, five were using statins, and three participants were using acetylsalicylic acid.

### 3.2. Sensor-Based Glucose Metrics

**Figure 1:**
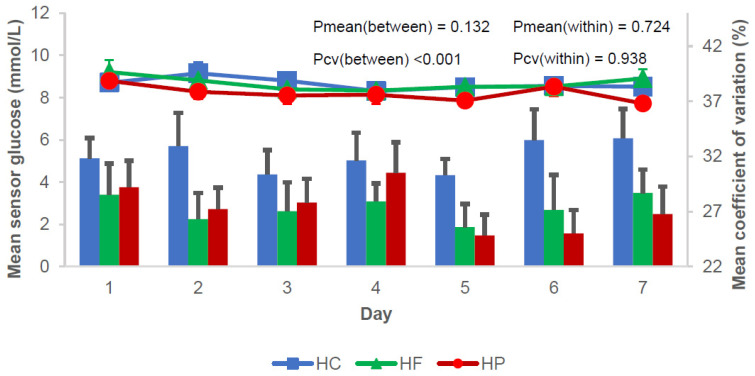
Mean sensor glucose and coefficient of variation for each day of the three diet weeks. Data are presented as mean ± SEM. *p*-values between and within are presented. HC, High-carbohydrate (Blue color); HF, Low-carbohydrate-high-fat (Green color); HP, Low-carbohydrate-high-protein (Red color).

Sensor-based glucose metrics are presented in Table 2 and Figure 1. Seven-day mean sensor glucose was similar between the diets (P_Overall_ = 0.08). Compared with HC, the two low-carbohydrate diets resulted in lower CV (Table 2).

The only significant difference between the two low-carbohydrate diets was time above range (TAR >10.0 mmol/L) (Table 2). On average, participants had 7.1 ± 3.2% less TAR during HP compared with HC and HF. The difference in TAR corresponded to one hour and forty minutes per day. TIR was significantly higher for HP compared with HC, whereas TBR was significantly lower for HF compared with HC (Table 2). Finally, seven participants achieved the composite glycemic target (TIR >70%, TAR <25%, and TBR <4%) during HP, compared with only three participants during HC and HF (Table 2).

All participants experienced ≥1 hypoglycemic event (a sensor glucose values <3.9 mmol/L) during HC. During HF and HP, four and three participants, respectively, had no sensor glucose values <3.9 mmol/L (Table 2). The number of hypoglycemic events lasting ≥15 min was significantly lower for HP compared with HC (Table 2). There were no events of severe hypoglycemia or diabetic ketoacidosis during the study.

### 3.3. Insulin

The total daily basal insulin dose was similar between diets (Table 3), while the total daily insulin bolus dose was significantly greater during the high-carbohydrate diet week compared with the two low-carbohydrate weeks (Table 3). The median (range) numbers of usages of extended or combined bolus per diet week were: HC: 0.5 (0–18), HF: 0.0 (0–17), and HP: 0.0 (0–17). 

### 3.4. Cardiovascular Risk Factors

The only significant difference in cardiovascular risk factors was a non-clinically relevant 0.5 kg difference in change in body weight between HC and HF (Table 3).

### 3.5. Protocol Adherence

The deviations between the carbohydrate recordings in the insulin pumps and the planned carbohydrate intake was minimal, suggesting acceptable adherence to all three study diets. Further, physical activity levels assessed by daily energy expenditures, metabolic equivalent of task rates, and step counts did not differ between diet weeks (Table 3).

## 4. Discussion

This study demonstrated that people with type 1 diabetes achieved a more favorable glucose profile when following an HF and especially an HP diet for one week compared with an isocaloric HC diet. There was no difference in mean glucose between diets, but variability was lower for the HF and HP diets compared with HC. The frequency of hypoglycemia events was less for HF and HP compared with HC. The only difference between the two low-carbohydrate diets was a lower TAR with HP. No clinically relevant differences in cardiovascular risk factors were seen. 

Despite the popularity of low-carbohydrate diets among people with type 1 diabetes, there is limited scientific evidence regarding their metabolic effects, especially with respect to the ratio between fat and protein [9,11,12,16]. The finding that low-carbohydrate diets reduce glycemic variability is in line with previous studies [13,14,15]. However, a novel observation was that the glucose stabilizing effect of carbohydrate restriction was consistent regardless of whether energy from carbohydrates was replaced by energy from fat or protein. Nonetheless, substitution with protein led to a greater reduction in TAR and there was a trend toward more people reaching the composite glycemic endpoint (TIR >70%, TAR <25%, and TBR <4%) during the HP than the HF intervention.

One explanation for the favorable impact of the HF and HP diets on glucose levels compared with the HC diet may be attributed to the differential responses to fat and protein in contrast to carbohydrates. Carbohydrate consumption rapidly elevates glucose levels post-absorption in the duodenum, whereas the effects of fats or proteins on glucose are intricate, resulting in a more gradual and delayed postprandial increase in glucose, particularly in late prandial hyperglycemia [20]. In the context of ingested fat, four mechanisms influencing glucose responses have been identified. First, fat absorption as triacylglycerol leads to glycerol formation, convertible to pyruvate which can be synthesized into glucose [21,22]. Second, free fatty acids in the bloodstream impact cellular responses to insulin, inducing increased insulin resistance [21,22]. Third, fat contributes to the release of glucose-regulating hormones, such as glucagon, glucagon-like protein 1, gastric inhibitor polypeptide, and ghrelin [20,22]. Finally, fat decreases the rate of gastric emptying [17]. These mechanisms collectively contribute to a slower glucose response to fat, causing delayed gastric emptying and subsequently slowing the glucose response to carbohydrates, when consumed in conjunction with fat [21]. Consequently, fat ingestion minimizes early glucose response (within the first 2–3 h) and delays peak glucose levels, leading to late post-prandial hyperglycaemia (>3 h) [17]. Regarding protein, two proposed mechanisms delineate its impact on glucose management [21]. Firstly, amino acids can be converted to glucose through gluconeogenesis [21,22]. Secondly, high protein intake stimulates the rise in hormones regulating glucose homeostasis, including glucagon, potentially resulting in hyperglycemia mediated by glycogenolysis and an increase in insulin resistance [22]. The ingestion of high-protein meals stimulates cortisol, growth hormone, insulin-like growth factor 1, and ghrelin, further contributing to delayed (2–3 h) and prolonged increase (>5 h) in glucose concentration [20,21,22].

When insulin is dosed according to carbohydrate content, the resulting insulin dynamics may match HP better than HF and this could explain the observed difference between the two low-carbohydrate diets. Previously, Smart et al. have demonstrated that the rise in glucose of a high-fat-low-protein meal was slower in the acute, post-prandial phase compared with a low-fat-high-protein meal (with equal carbohydrate content) explained by delay in gastric emptying due to the high-fat content [20].

To diminish late postprandial hyperglycemia after high-fat or high-protein meals, a larger insulin dose may be needed compared with lower fat/protein meals with identical carbohydrate content [17]. Nevertheless, in this study, we did not provide specific bolus guidance, i.e., we did not encourage insulin dosing for fat and protein nor the use of extended bolus and combined bolus, and the use of other bolus types than immediate delivery was low. We also did not give directions regarding the time interval between bolus delivery and meal initiation, and we did not adjust the basal insulin rates prior to each diet week. Use of different bolus types, modification of basal rates, and time between bolus and meal start during the different diet interventions could potentially have affected the observed differences in glucose outcomes. On the other hand, the chosen approach showed that it is possible to apply different diet strategies at different time points without need for the adjustment of insulin pump settings. 

In line with the current study, Dimosthenopoulos et al. showed positive effects of one week of a high-protein diet (energy provided by carbohydrate: 20%, fat: 40%, protein 40%)), demonstrating that people spent less time in hypoglycemia and had lower glycemic variability compared with a reference diet (energy provided by carbohydrate: 50%, fat: 30% and protein 20%) [15]. Likewise, single meal studies with meals high in protein have shown lower mean postprandial glucose excursions, lower glycemic variability, and less drop in glucose during a subsequent exercise session [23,24]. This, together with the current study, suggest that maximizing dietary protein content within the recommended limits, i.e., 1.2–1.8 g per kilo body weight per day, might be particularly beneficial for blood glucose management in type 1 diabetes. 

In relation to low-carbohydrate diets, concerns about the risk of hypoglycemia and diabetic ketoacidosis have been raised [9]. However, the numbers of mild hypoglycemia events (sensor glucose <3.9 mmol/L) during HP and HF were numerically lower than during HC, and HP significantly reduced the number of hypoglycemia events per participant lasting 15 min or more compared with HC. No severe adverse events of any kind were registered.

While our glucose results stand strong, the effects of the three diets on cardiovascular risk factors should be interpreted with caution due to the short study duration. Apart from a minor difference in change in body weight between HC and HF, we did not see any significant effect on lipids or blood pressure during either low- or high-carbohydrate diets. Whether longer term exposure to the different diet types would affect cardiovascular risk remains to be determined. However, in overweight people with and without type 2 diabetes, cardiovascular risk markers remained unchanged for up to two years after the introduction of a low-carbohydrate diet [25]. 

A strength of this study was the individually tailored diets, which may have contributed to the relatively high adherence to the protocol. However, a limitation of the design was the absence of complete control over and insight into the participants’ actual food consumption. The inclusion of insulin pump and isCGM/CGM users only enabled collection of detailed information regarding insulin doses and glucose values. These individuals represent a selected group of people with type 1 diabetes; nevertheless, it is expected that people treated with insulin injections may derive comparable benefits from HF and HP dietary strategies. Since there is currently little evidence supporting different low-carbohydrate strategies in type 1 diabetes, we aimed to demonstrate the feasibility and proof-of-concept in a small and short-term study. Larger and longer-lasting studies are needed to uncover all the positive and negative effects of low-carbohydrate diets. 

## 5. Conclusions

In summary, this study demonstrated that adults with type 1 diabetes experienced a more advantageous glucose profile during one-week of HF or HP compared with an HC diet. Specifically, HP optimized the sensor-based glucose metrics. No adverse events were observed for any of the diets. The study underlines the importance of considering overall macronutrient composition, in addition to the carbohydrate load, for people with type 1 diabetes. 

## Figures and Tables

**Table 1:** Diet compositions (in grams and energy percentages) for a 70 kg person with a calorie need of 30 Kcal/kg body weight/day.

Diet Type	Carbohydrate	Fat	Protein
High-carbohydrate (HC)	250 g	78 g	98 g
	(48%)	(33%)	(19%)
Low-carbohydrate-high-fat (HF)	100 g	145 g	98 g
	(19%)	(62%)	(19%)
Low-carbohydrate-high-protein (HP)	100 g	132 g	126 g
	(19%)	(57%)	(24%)

HC, High-carbohydrate; HF, Low-carbohydrate-high-fat; HP; Low-carbohydrate-high-protein.

**Table 2:** Sensor-based glucose metrics.

*n* = 12	HC	HF	HP	*p*-Value (Overall)	*p*-Value (HC vs. HF)	*p*-Value (HC vs. HP)	*p*-Value (HF vs. HP)
Mean sensor glucose (mmol/L)	8.7 ± 1.0	8.7 ± 1.1	8.2 ± 1.0	0.08	-	-	-
Standard deviation (mmol/L)	3.0 ± 0.5	2.7 ± 0.7	2.5 ± 0.6	0.002 *	0.042 *	0.002 *	0.371
Coefficient of variation (%)	34.5 ± 4.1	30.5 ± 6.2	30.0 ± 5.5	0.002 *	0.009 *	0.003 *	0.906
Time in 3.9–10.0 mmol/L (%)	67.5 ± 13.1	69.2 ± 11.5	75.8 ± 11.5	0.041 *	0.853	0.043 *	0.126
Time in 3.9–7.8 mmol/L (%)	42.4 ± 12.4	41.7 ± 16.0	48.5 ± 16.1	0.157	-	-	-
Time below 3.9 mmol/L (%)	3.0 ± 2.7	1.3 ± 1.1	1.9 ± 1.9	0.022 *	0.019 *	0.140	0.601
Time below 3.0 mmol/L (%)	0.5 ± 0.7	0.1 ± 0.2	0.2 ± 0.5	0.148	-	-	-
Time above 10.0 mmol/L (%)	29.5 ± 13.4	29.4 ± 12.1	22.3 ± 11.8	0.019 *	1.0	0.037 *	0.037 *
Time above 13.9 mmol/L (%)	6.9 ± 4.9	4.6 ± 5.4	3.1 ± 3.7	0.035 *	0.230	0.007 *	0.222
Participants achieving glycemic targets † (number)	3	3	7	0.033 *	1.0	0.057	0.057
Participants experiencing ≥1 hypoglycemia events (sensor glucose <3.9 mmol/L) during diet week (number)	12	8	9	-	-	-	-
Hypoglycemia events lasting ≥15 min per participant per week (number)	5.4	3.1	2.6	0.015 *	0.074	0.019 *	0.873

Data are presented as mean ± SD. * *p*-value <0.05. † Time in 3.9–10.0 mmol/L >70%, time above 10.0 mmol/L <25%, and time below 3.9 mmol/L <4%. HC, High-carbohydrate; HF, Low-carbohydrate-high-fat; HP, Low-carbohydrate-high-protein.

**Table 3:** Cardiovascular risk factors, insulin, and protocol adherence.

*n* = 12	HC	HF	HP	*p*-Value (Overall)	*p*-Value (HC vs. HF)	*p*-Value (HC vs. HP)	*p*-Value (HF vs. HP)
Cardiovascular risk factors							
∆ Body weight (kg)	−0.2 (−0.8; 0.5)	−1.5 (−2.3; −0.7)	−1.0 (−1.4; −0.5)	0.004 *	0.003 *	0.072	0.37
∆ Systolic blood pressure (mmHg)	−3 (−13; 2)	2 (−2; 7)	−1 (−6; 3)	0.37	-	-	-
∆ Diastolic blood pressure (mmHg)	0 (−3; 3)	2 (−2; 4)	1 (−8; 4)	0.88	-	-	-
∆ Heart rate (bpm)	0 (−4; 3)	−4 (−9; −1)	−3 (−6; −1)	0.10	-	-	-
%-change UACR †	6.2 (−32.0; 58.0)	−14.8 (−40.0; 50.7)	−16.5 (−42.3; 0.0)	0.52	-	-	-
∆ eGFR † (mL/min/1.73 m^2^)	−2.6 (0.0; 1.5)	−1.0 (−1.0; 0.0)	−2.5 (−3.0; 0.0)	0.48 ^NP^	-	-	-
∆ Total cholesterol (mmol/L)	−0.5 (−0.8; −0.3)	−0.2 (−0.3; −0.1)	−0.1 (−0.5; 0.3)	0.11	-	-	-
∆ HDL (mmol/L)	−0.3 (−0.4; 0.0)	−0.1 (−0.5; 0.2)	−0.2 (−0.3; −0.1)	0.22	-	-	-
∆ LDL (mmol/L)	−0.2 (−0.4; 0.0)	0.0 (−0.2; 0.1)	0.1 (−0.2; 0.5)	0.11	-	-	-
∆ VLDL (mmol/L)	−0.0 (−0.1; 0.0)	−0.0 (−0.1; 0.0)	−0.2 (−0.1; 0.0)	0.38 ^NP^	-	-	-
∆ Triglycerides (mmol/L)	−0.1 (−0.1; 0.1)	−0.1 (−0.4; 0.0)	−0.2 (−0.5; 0.3)	0.75 ^NP^	-	-	-
Insulin							
Total daily basal insulin (U)	20.6 (12.4; 22.4)	20.7 (12.6; 22.0)	20.7 (12.6; 22.8)	0.91	-	-	-
Total daily bolus insulin (U)	29.1 (19.7; 32.3)	14.5 (8.4; 17.3)	13.5 (8.2; 14.8)	<0.001 *	<0.001 *	<0.001 *	0.92
Total daily insulin dose (U)	49.8 (32.6; 52.9)	35.2 (26.0; 34.6)	34.2 (22.1; 35.6)	<0.001 *	<0.001 *	<0.001 *	0.68
Deviations from planned carbohydrate intake based on insulin pump entries							
Deviation (g)	−4.8 (−25.3; 9.9)	10.7 (−3.1; 28.4)	5.7 (−1.2; 2.5)	0.14	-	-	-
Deviation (%)	12.0 (2.2; 21.0)	18.7 (3.7; 36.5)	12.7 (0.8; 19.1)	0.14	-	-	-
Activity level							
Energy expenditure per day (kcal) ‡	1481 (1220; 1752)	1591 (1248; 1769)	1563 (1184; 1855)	0.70	-	-	-
MET rate per day ‡	1.49 (1.40; 1.60)	1.53 (1.39; 1.62)	1.52 (1.40; 1.62)	0.70	-	-	-
Steps per day ‡	12,552 (9306; 15,374)	12,815 (10,693; 16,258)	12,908 (10,399; 15,597)	0.90	-	-	-

Data are presented as mean (IQR). * *p*-value < 0.05. † Geometric means. ‡ ActiGraph-derived measurements. eGFR, Estimated glomerular filtration rate; HC, High-carbohydrate; HDL, High-density lipoprotein; HF; Low-carbohydrate-high-fat; HP, Low-carbohydrate-high-protein; IQR, Interquartile range; LDL, Low-density lipoprotein; MET, Metabolic equivalent of task; NP, Non-parametric test; *p*, *p*-value; UACR, Urine albumin–creatinine ratio; VLDL, Very-low-density lipoprotein.

## Data Availability

The data presented in this study are available from the corresponding author upon reasonable request. The data are not publicly available due to data protection rules.

## Supplementary Materials

The following supporting information can be downloaded at: https://www.mdpi.com/article/10.3390/nu16020199/s1, Figure S1: Flow diagram. HC, High-carbohydrate; HF, Low-carbohydrate-high-fat; HP, Low-carbohydrate-high-protein; Table S1: Example of a high-carbohydrate diet plan; Table S2: Example of a low-carbohydrate-high-fat diet plan; Table S3: Example of a low-carbohydrate-high-protein diet plan.
