# Supplementary material for: Sensor-Based Glucose Metrics during Different Diet Compositions in Type 1 Diabetes—A Randomized One-Week Crossover Trial
Source: Nutrients. 2024 Jan 8;16(2):199. doi: 10.3390/nu16020199 (PMC10820230; doi:10.3390/nu16020199)
Supplement: Supplementary file 1 [file nutrients-16-00199-s001.zip › nutrients-2738418-supplementary.pdf]

nutrients MDPI

Supplementary Materials

Table S1. Example of a high-carbohydrate diet plan.

	Amount	Total (g)	Energy (Kcal)	Carbohydrates (g)	Fat (g)	Protein (g)
**Breakfast**		**1405**	**513**	**76**	**9**	**25**
Oats	3 dl	105	387	61	7	14
Skimmed milk (0.1%)	3 dl	300	113	14	2	11
Coffee	5 cups (20 cl)	1000	12	1	0	1
**Lunch**		**492**	**774**	**52**	**46**	**35**
Salmon		110	195	0	12	22
Potatoes		180	147	30	1	3
Rye bread (whole grain)	1 slice	45	91	17	1	2
Cooking oil	2 tbsp	27	239	0	27	0
Vegetable garnish blend (frozen)		100	26	4	1	2
Feta (40+)		30	76	0	6	6
**Afternoon**		**170**	**257**	**39**	**6**	**8**
Rye bread (whole grain)	1 slice	45	91	17	1	2
Cheese (hard, 45+)	1 slice	20	67	0	5	5
Banana (medium size)	1	105	99	22	0	1
**Dinner**		**875**	**867**	**55**	**50**	**46**
Chicken (meat, raw)		150	193	0	8	29
Potatoes		180	147	30	1	3
Cooking oil	2 tbsp	27	239	0	27	0
Vegetable garnish blend (frozen)		100	26	4	1	2
Sun-dried tomatoes in oil	3 pcs	18	38	3	3	1
Black olives (pitted, in brine)	20 pcs	100	111	3	10	1
Skimmed milk (0.5%)	3 dl	300	113	14	2	11
**Evening**		**230**	**386**	**40**	**12**	**26**
Rye bread (whole grain)	2 slices	90	181	34	1	5
Cheese (hard, 45+)	2 slices	40	133	1	10	10
Skyr (0.2%)		100	71	6	0	11
**Total**		**3172**	**2796**	**262**	**123**	**140**
180 g potatoes alternatives		**852**	**1183**	**214**	**8**	**48**
Pasta (fresh, boiled)		*112*	*147*	*28*	*1*	*5*
Rice (boiled)		*112*	*146*	*32*	*0*	*3*
Rye bread (whole grain)		*75*	*153*	*29*	*1*	*4*
Whole-grain bread		*68*	*148*	*36*	*2*	*9*
Quinoa (boiled)		*90*	*146*	*23*	*2*	*6*
Bulgur (boiled)		*180*	*149*	*25*	*0*	*6*
Red lentils (boiled)		*100*	*148*	*23*	*1*	*12*
Kidney beans (boiled)		*115*	*146*	*18*	*1*	*10*

Table S2. Example of a low-carbohydrate-high-fat diet plan.

	Amount	Total (g)	Energy (Kcal)	Carbohydrates (g)	Fat (g)	Protein (g)
**Breakfast**		**1261**	**358**	**18**	**26**	**12**
Greek yogurt (10%)		250	308	11	25	10
Oats	2 tbsp	11	39	6	1	1
Coffee	5 cups (20 cl)	1000	12	1	0	1
**Lunch**		**422**	**725**	**20**	**53**	**44**
Salmon		175	310	0	19	35
Potatoes		90	74	15	0	2
Cooking oil	2 tbsp	27	239	0	27	0
Vegetable garnish blend (frozen)		100	26	4	1	2
Feta (40+)		30	76	0	6	6
**Afternoon**		**130**	**392**	**18**	**25**	**22**
Rye bread (whole grain)	1 slice	45	91	17	1	2
Butter (salted)	1 tsp	5	35	0	4	0
Cheese (hard, 45+)	4 slices	80	267	1	21	20
**Dinner**		**600**	**814**	**30**	**53**	**51**
Chicken (meat, raw)		235	302	0	13	45
Potatoes		120	98	20	0	2
Cooking oil	2 tbsp	27	239	0	27	0
Vegetable garnish blend (frozen)		100	26	4	1	2
Sun-dried tomatoes in oil	3 pcs	18	38	3	3	1
Black olives (pitted, in brine)	20 pcs	100	111	3	10	1
**Evening**		**370**	**485**	**14**	**40**	**16**
Greek yogurt (10%)		250	308	11	25	10
Cheese (hard, 45+)	1 slice	20	67	0	5	5
Black olives (pitted, in brine)	20 pcs	100	111	3	10	1
**Total**		**2782**	**2774**	**100**	**197**	**145**
120 g potatoes alternatives		***570***	***792***	***143***	***5***	***32***
Pasta (fresh, boiled)		*75*	*98*	*19*	*1*	*4*
Rice (boiled)		*75*	*98*	*21*	*0*	*2*
Rye bread (whole grain)		*50*	*102*	*20*	*1*	*3*
Whole-grain bread		*45*	*98*	*24*	*1*	*2*
Quinoa (boiled)		*60*	*97*	*15*	*2*	*4*
Bulgur (boiled)		*120*	*100*	*17*	*0*	*4*
Red lentils (boiled)		*70*	*103*	*16*	*1*	*8*
Kidney beans (boiled)		*75*	*95*	*12*	*0*	*7*

Table S3. Example of a low-carbohydrate-high-protein diet plan.

	Amount	Total (g)	Energy (Kcal)	Carbohydrates (g)	Fat (g)	Protein (g)
**Breakfast**		**1295**	**437**	**27**	**19**	**35**
Skyr (0.2%)		200	131	9	0	22
Rye bread (whole grain)	1 slice	45	91	17	1	2
Butter (salted)	2 tsp	10	69	0	8	0
Cheese (hard, 45+)	2 slices	40	133	1	10	10
Coffee	5 cups (20 cl)	1000	12	1	0	1
**Lunch**		**497**	**829**	**25**	**58**	**53**
Salmon		220	390	0	24	44
Potatoes		120	98	20	0	2
Cooking oil	2 tsp	27	239	0	27	0
Vegetable garnish blend (frozen)		100	26	4	1	2
Feta (40+)		30	76	0	6	6
**Afternoon**		**80**	**267**	**1**	**21**	**20**
Cheese (hard, 45+)	4 slices	80	267	1	21	20
**Dinner**		**665**	**897**	**30**	**57**	**64**
Chicken (meat, raw)		300	385	0	17	58
Potatoes		120	98	20	0	2
Cooking oil	2 tbsp	27	239	0	27	0
Vegetable garnish blend (frozen)		100	26	4	1	2
Sun-dried tomatoes in oil	3 pcs	18	38	3	3	1
Black olives (pitted, in brine)	20 pcs	100	111	3	10	1
**Evening**		**340**	**375**	**12**	**21**	**33**
Skyr (0.2%)		200	131	9	0	22
Cheese (hard, 45+)	2 slices	40	133	1	10	10
Black olives (pitted, in brine)	20 pcs	100	111	3	10	1
**Total**		**2877**	**2805**	**95.7**	**175**	**205**
120 g potatoes alternatives		***570***	***792***	***143***	***5***	***32***
Pasta (fresh, boiled)		*75*	*98*	*19*	*1*	*4*
Rice (boiled)		*75*	*98*	*21*	*0*	*2*
Rye bread (whole grain)		*50*	*102*	*20*	*1*	*3*
Whole-grain bread		*45*	*98*	*24*	*1*	*2*
Quinoa (boiled)		*60*	*97*	*15*	*2*	*4*
Bulgur (boiled)		*120*	*100*	*17*	*0*	*4*
Red lentils (boiled)		*70*	*103*	*16*	*1*	*8*
Kidney beans (boiled)		*75*	*95*	*12*	*0*	*7*

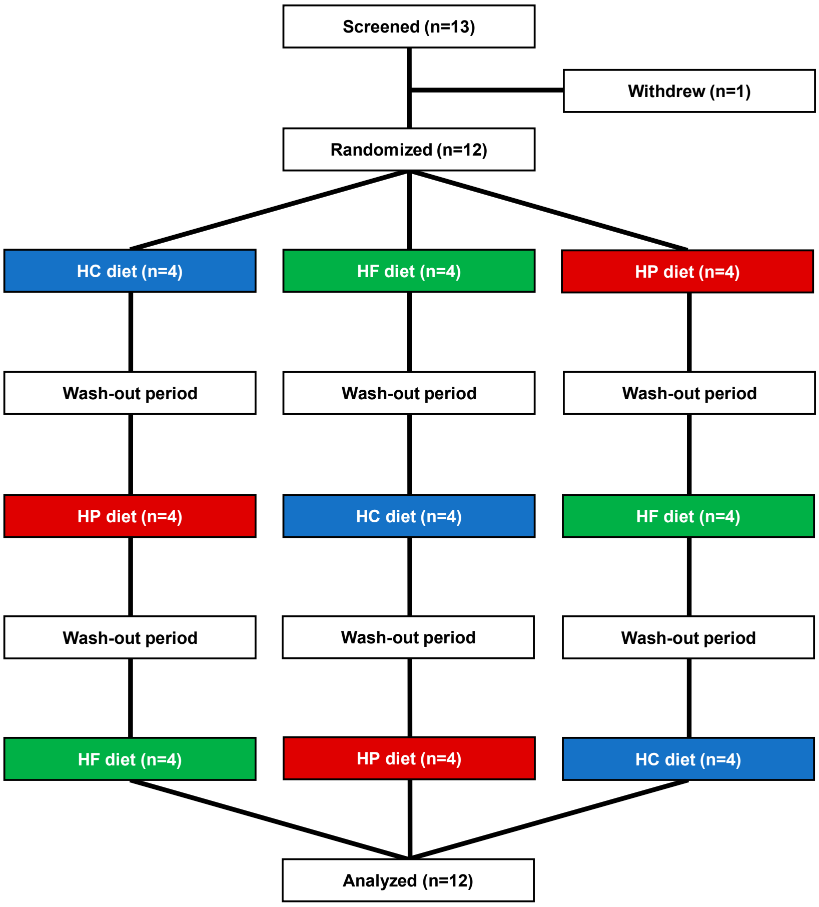

Figure S1. Flow diagram. HC, High-carbohydrate; HF, Low-carbohydrate-high-fat; HP, Low-carbohydrate-high-protein.
